# Assessing the feasibility of injectable growth-promoting therapy in Crohn’s disease

**DOI:** 10.1186/s40814-016-0112-9

**Published:** 2016-12-05

**Authors:** Mabrouka A. Altowati, Ashley P. Jones, Helen Hickey, Paula R. Williamson, Farah M. Barakat, Nicolene C. Plaatjies, Ben Hardwick, Richard K. Russell, Thomas Jaki, S. Faisal Ahmed, Ian R. Sanderson

**Affiliations:** 1 Developmental Endocrinology Research Group, University of Glasgow, Scotland, UK; 2 Medicines for Children Clinical Trials Unit, Clinical Trial Research Centre, University of Liverpool, Liverpool, UK; 3 Centre for Digestive Diseases, Blizard Institute, Barts and The London School of Medicine and Dentistry, Queen Mary University of London, 4 Newark Street, London, E1 2AT UK; 4 Department of Paediatric Gastroenterology, Royal Hospital for Sick Children, Glasgow, UK; 5 Medical and Pharmaceutical Statistics Research Unit, Department of Mathematics and Statistics, Lancaster University, Lancaster, UK

**Keywords:** Growth retardation, Inflammatory bowel disease, Crohn’s disease, Puberty, Growth hormone, Insulin-like growth factor-1

## Abstract

**Background:**

Despite optimal therapy, many children with Crohn’s disease (CD) experience growth retardation.

The objectives of the study are to assess the feasibility of a randomised control trial (RCT) of injectable forms of growth-promoting therapy and to survey the attitudes of children with CD and their parents to it.

**Methods:**

A feasibility study was carried out to determine study arms, sample size and numbers of eligible patients. A face-to-face questionnaire surveyed willingness to consent to future participation in the RCT. Eligibility to the survey was any child under 18 (with their parent/guardian) with CD whose height standard deviation score (HtSDS) was ≤+1. Of 118 questionnaires, 94 (80%) were returned (48 by children and 46 by parents).

**Results:**

The median age of the patients in the survey was 14.3 years (range 7.0 to 17.7), and 35 (73%) were male. Their median HtSDS was −1.2 (−3.01, 0.23), and it was lower than the median mid-parental HtSDS of −0.6 (−3.1, 1.4). We analysed the willingness of the children whose HtSDS <−1 to take part in the proposed RCT, being those most likely to require treatment. Overall, 18 (47%) children and 17 (46%) parents were willing. This increased to 61% of children who were slightly concerned about their height and 100% (4/4) of those very concerned. A common reason for not taking part in the RCT was fear of injections (44%); 111 children are required for randomisation into three study arms from nine centres.

**Conclusions:**

Almost half of children and parents surveyed would take part in an RCT of growth-promoting therapy. Allaying fears about injections may result in higher recruitment rates.

**Electronic supplementary material:**

The online version of this article (doi:10.1186/s40814-016-0112-9) contains supplementary material, which is available to authorized users.

## Background

Around one quarter of cases of Crohn’s disease (CD) are diagnosed in children under 18, with the incidence in childhood increasing [1]. Growth failure is a common manifestation and may be the first presentation of disease [2]. Despite advances in CD treatments [3], around 20% of affected children may continue to grow slowly [4] and remain short on reaching their final adult height [5, 6].

The cause of growth failure is multi-factorial and includes poor nutrition, inflammation and corticosteroid treatment. The mechanisms involve a disturbance of growth hormone insulin-like growth factor-1 axis (GH-IGF-1) at peripheral and central levels [7]. Although the exact mechanisms of disturbance of the GH-IGF-1 axis in CD are still not fully elucidated, the abnormalities may range from functional GH deficiency to GH resistance with low circulating IGF-1 [7–10]. Current treatment is to improve growth using steroid-sparing anti-inflammatory medication [11].

Preliminary evidence from the use of recombinant growth hormone (rhGH) therapy in CD children in our centres and from centres in the USA indicates its potential efficacy despite the expected insensitivity to GH [12–14]. However, there are no randomised control trials (RCTs) that investigate recombinant human insulin-like growth factor-1 (rhIGF-1) alone or in combination with rhGH on growth of children with CD. As GH acts through IGF-1 and because children with Crohn’s disease have low circulating IGF-1, rhIGF-1 is worthy of consideration, being efficacious in stimulating growth in children with conditions associated with GH insensitivity (reviewed in [10]).

It is, therefore, possible that the use of rhGH and/or rhIGF-1 might enhance the growth of children with Crohn’s disease with growth failure, over and above that achieved with optimal anti-inflammatory therapy. The rationale for conducting a future study was to determine if we could develop a treatment strategy to combat growth failure in children with Crohn’s disease. There are, however, concerns with regard to the acceptability of conducting such a trial to both patients with CD and their families. It is important to establish whether it is feasible to conduct a trial of this nature. We, therefore, undertook a feasibility study to:

- Survey the attitudes of parents and patients towards the proposed RCT
- Establish an experimental design (including the number of arms with different treatments)
- Estimate the number of patients with CD who would potentially be available from UK centres
- Identify suitable sites and collaborators
- Establish the required sample size

## Methods

We applied to the appropriate Medicines for Children Clinical Studies Group (CSG) of the National Institute for Health Research (NIHR) whose remit is to help the NIHR define research priorities and to develop a portfolio of clinical studies to be run across the NIHR Medicines for Children network. It is the purpose of the CSG to determine if a question is scientifically valid and clinically important. It includes parent and patient involvement. We obtained a satisfactory outcome to examine the feasibility of a trial of injectable growth-promoting drugs with a view to establishing an RCT, before applying for funds from Crohn’s in Childhood Research Association (CICRA). CICRA undertook peer review using standards approved by the Association Medical Research Charities (AMRC).

The Medicines for Children’s Clinical Trials Unit (MC CTU) administered the feasibility study which included 10 monthly trial management group (TMG) meetings either by teleconference or face to face. Minutes of these meetings were recorded and made available to all members.

### Patient survey

Two questionnaire surveys were developed for two groups: potential participants for the trial (Additional file 1) and their parents (Additional file 2). These questionnaires were developed by the TMG, and their format was based on a successful earlier questionnaire survey by the MC-CTU [15]. As the questionnaire was a survey on participants’ willingness to consent to a future trial, it did not require research ethics committee approval, as long as the answers to the questionnaires remained anonymous, as determined by the Barts Health NHS Trust R&D office. Anonymity was important to avoid the possibility of parents and children giving different answers because they thought that their healthcare provider could see and identify them. Information sheets were written for the clinical teams, which included a description of the four possible arms of the RCT: (1) treatment to combat inflammation, (2) added injections of recombinant human growth hormone (rhGH), (3) added injections of recombinant human insulin-like growth factor (rhIGF), and (4) added rhGH and rhIGF-1.

Content validity was assessed through questionnaires piloted on five patients and their parents. They were amended based on feedback and then sent to a wider group. The questions (Additional files 1 and 2) included demographic data: age, height on the day of attending clinic and parental height. Specific questions included their degree of concern regarding their height, attitudes to injections to improve growth, willingness to join an RCT and any previous growth-promoting drugs. All questionnaires were anonymous, and no patient identifiable data were collected. Parents and patients in the same family were given a single study number to enable comparison of responses.

### Participants

Questionnaires were distributed in two paediatric inflammatory bowel disease (IBD) outpatient clinics (Barts Health (London) and Royal Hospital for Sick Children (Glasgow)) to consecutive participants between 1 March and 31 July 2014. One further centre that was invited did not administer questionnaires because of limitations in research capacity. The target recruitment at each of the centres was at least 30 patient questionnaires and 30 parent questionnaires. The child questionnaire was completed by a patient with CD who fulfilled the eligibility criteria (height standard deviation score (HtSDS) was ≤+1), and only the 38 children with HtSDS ≤−1 were included in the attitude analysis because only children whose HtSDS was <−1 were to be included in the RCT (see below). One of the child’s parents completed the parental questionnaire. In two children who participated, the parents did not answer a questionnaire. We, therefore, analysed questionnaires from 38 children and 36 parents for the attitude analysis. A healthcare worker was available to explain the meaning of any questions that a child or parent did not understand but was not involved in recording the answers.

Completed questionnaires were sent back to the MC CTU for initial result collation. For the study participants, height was measured with a Harpenden stadiometer and converted into standard deviation scores (SDS) for chronological age using 1990 UK standards [16]. The HtSDS is the number of standard deviations that a particular child deviates above (+) or below (−) the mean for that child’s age. Mid-parental height (MPH) and MPH SDS were calculated from reported parenteral heights.

### Survey of paediatric gastroenterologists

A letter was sent to 18 paediatric IBD centres in the UK and to 6 general paediatric centres with an interest in gastroenterology. These explained the feasibility study in terms of a possible future RCT. It also asked for the numbers of children with Crohn’s disease managed under their care. A 2-week timeline was given to receive replies.

### Analysis

Data were analysed using Minitab software version 17 and SAS (version 9.2). Non-parametric data are presented with medians and ranges. For categorical variables, percentages were calculated. Missing responses were not included in the descriptive analyses.

## Results

The eligibility was determined in two centres (Barts Health and Glasgow), by studying children with CD who had severe growth failure (HtSDS <−1 combined with a reduction in HtSDS or 0.5 more over 1 year).

### Survey results

Willingness to consent was surveyed using questionnaires in children with short stature (HtSDS <+1). This number included a wider range than our proposed treatment group. This would allow us to determine if height might affect a participant’s willingness to undertake the study. However, in order to more closely align our results with a proposed RCT, we analysed the willingness of only those children with an HtSDS <−1. Nevertheless, it was not possible to make a reduction of 0.5 HtSDS a criterion for inclusion into the survey because that would have lost anonymity: although a static HtSDS can be calculated from an anonymous survey (parents know the age of their child, and the height measured in outpatients), calculating a change in HtSDS over a year requires access to medical records (which are not anonymous) to determine earlier heights and their dates of measurement.

The overall response rate was 78% (48 (80%) out of 60 questionnaires were completed by children and 46 (77%) out of 60 were completed by parents). Participants’ demographics, anthropometric and clinical information are presented in Table 1. Of the 48 children, 4 (9%) had been specifically treated with a growth-promoting therapy before (*n* = 2 received rhGH and *n* = 2 received testosterone). The responses from children and parents to the survey’s questions are summarised in Table 2. In 31 cases, there was agreement with regard to how concerned a child and their parent were with the child’s height.

**Table 1:** Demographic, anthropometry and clinical characteristics

	Total *n* = 48 (80%)
Age/year (range)	14.3 (7.0, 17.7)
Sex (M), *n* (%)	35 (73)
HtSDS (range)	−1.2 (−3.01, 0.89)
MPHSDS (range)	−0.59 (−3.14, 1.4)
Treated for growth problem, *n* (%)	4 (8)
Family history of CD, *n* (%)	8 (17)

*MPHSDS* mid-parental height SDS, *HtSDS* height SDS score, *CD* Crohn’s disease

**Table 2:** The responses from children and parents to survey’s questions

		Total		
Question		Response	Parents (*n* = 46)	Child (*n* = 48)
1	How concerned are you about your child’s height?	Not concerned	29 (63%)	26 (54%)
		Slightly concerned	11 (24%)	19 (40%)
		Very concerned	5 (11%)	3 (6%)
		Missing	1 (2%)	
2	Do you think it is worth doctors trying to find a better treatment for growth in Crohn’s disease?	Yes	40 (87%)	42 (88%)
		No	4 (9%)	5 (10%)
		Missing	2 (4%)	1 (2%)
3	Do you think that the opportunity of gaining extra height is worth a year of daily injections?	Yes	25 (54%)	20 (41%)
		No	19 (41%)	28 (58%)
		Missing	2 (4%)	0
4	We have explained that in an RCT you are not able to choose which treatment your child would receive. Would you be comfortable with this?	Yes	20 (44%)	24 (50%)
		No	25 (54%)	23 (47%)
		Missing	1 (2%)	1 (2%)
5	Would you and your child be willing to attend to have your child’s growth and other things checked (e.g. quality of life) if it sometimes means an extra visit (1 or 2 extra in a year)?	Yes	34 (74%)	36 (75%)
		No	10 (22%)	12 (25%)
		Missing	2 (4%)	0
6	If the RCT we had in mind was happening now, would you be willing for your child to join?	Yes	22 (48%)	21 (44%)
		No	23 (50%)	27 (56%)
		Missing	1 (2%)	0

The results of cross-tabulation of question 1 (How concerned are you about your/your child’s height?) and question 6 (If the RCT we had in mind was happening now, would you be willing for your child to join?) are shown in Tables 3 and 4 for participants with HtSDS <−1, for children and their parents, respectively; 11/38 (47%) of children were willing to participate in a future RCT. Although 4/17 (23%) patients were not concerned about their height, they were willing to participate [median HtSDS (range) for patients who were willing, −1.3 (−2.2 to −1.0), and not willing, −1.1 (−1.5 to −1.0), to participate in the RCT]. Furthermore, 11/18 (61%) children who were slightly concerned about their height responded that they would be happy to participate in the RCT [median (range) HtSDS was −1.6 (−3.0 to −1.0) compared to 7/18 (39%) who were slightly concerned and not willing to participate, −1.3 (−1.95 to −1.0)]. All very concerned children were willing to take part if the RCT went forward [median (range) HtSDS −2.1 (−2.5 to −1.2)]. In summary, therefore, at least 60% of children who showed concern about their height would be willing to take part in an RCT of injectable therapy.

**Table 3:** Children’s willingness to participate. Cross-tabulation of questions was examined to understand if a child’s attitude to their height influenced their willingness to have injectable treatment: question 1 (How concerned are you about your height?) was compared to question 6 (If the RCT we had in mind was happening now, would you be willing to join?) for children (*n* = 38)

		Question 6 If the RCT we had in mind was happening now, would you be willing to join?	
		Total	
Question 1 How concerned are you about your height?		No	Yes
	Not concerned *n* (%)	13 (76%)	4 (23%)
	Slightly concerned *n* (%)	7 (39%)	11 (61%)
	Very concerned *n* (%)	0 (0%)	3 (100%)

**Table 4:** Parents’ willingness to participate. Cross-tabulation of question 1 (How concerned are you about your child’s height?) and question 6 (If the RCT we had in mind was happening now, would you be willing for your child to join?) for parents (*n* = 36) (missing data for relevant questions from returned questionnaires = 1)

		Question 6 If the RCT we had in mind was happening now, would you be willing for your child to join?	
		Total	
Question 1 How concerned are you about your child’s height?		No	Yes
	Not concerned *n* (%)	11 (58%)	8 (42%)
	Slightly concerned *n* (%)	4 (36%)	7 (64%)
	Very concerned *n* (%)	1 (20%)	4 (80%)

Although 8/19 (42%) parents were not concerned about their children’s growth, they were willing to have their children participate in the RCT. The median (range) HtSDS of these children was −1.4 (−3.0 to −1.0), compared to −1.4 (−2.0 to −1.0) for children of the 11/19 (58%) parents who were not concerned and not willing to participate in the RCT. In contrast, 7/11 (64%) of parents who were slightly concerned were willing for their children to join the RCT. The median (range) HtSDS [−1.4 (−2.3 to −1.0) vs. −1.2 (−1.6 to −1.0), respectively] of these two groups were similar. In addition, 4/5 (80%) of very concerned parents were willing for their children to participate in the RCT [median (range) HtSDS −1.8 (−2.5 to −1.1)].

**Fig. 1:**
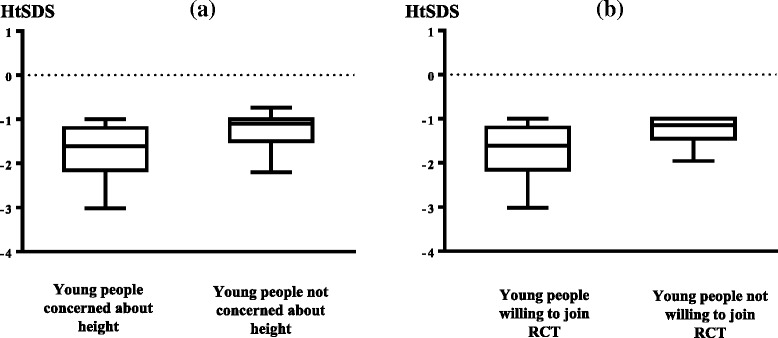
Differences in HtSDS between children who were concerned and those who were not concerned about their height (**a**) and difference in HtSDS between children who are willing to participate in randomised controlled trial and those who are not willing (**b**). *HtSDS* height SDS

The median (range) HtSDS [−1.5 (−3.01 to −1.0)] in the concerned children was lower than that in the non-concerned group [median (range) HtSDS −1.1 (−2.2 to −1.0)] (Fig. 1a). Their gender distribution, however, was found to be similar (15 M/6 F vs. 12 M/5 F). Also, the MPHSDS [−0.7 (−3.1 to 0.54) vs. −0.7 (−2.2 to 0.63)] among concerned and non-concerned children were similar. The 18/38 (47%) children who were willing to participate in the RCT were shorter [median (range) with a HtSDS −1.6 (−3.0 to −1.0)] than the 20 (53%) who were not willing to participate in RCT [median (range) HtSDS −1.1 (−1.9 to −1.0)] (Fig. 1b; but, these two groups were similar with respect to their gender (12 M/6 F vs. 15 M/5 F)).

The major reasons for not wishing to participate in the RCT were identified by 18 children: 8 (44%) of them stated the fear of injections, 8 (44%) stated that they were not concerned about their height, 1 (6%) participant was already on many drugs and 1 (6%) difficulty in taking time off from college.

### Study design including number of arms

During the period of the feasibility study, the availability of rhIGF-1 for research was discontinued, due to manufacturing problems and the need to conserve stocks for children in clinical need. This necessitated a complete change in study design, as two of the arms, originally envisaged, included rhIGF-1. We therefore chose a three-arm design with two rhGH doses. The doses were chosen for the following reasons: one dose was that used in our preliminary study [14] and the second dose was that used clinically to stimulate growth in other chronic diseases of childhood such as renal failure and cystic fibrosis. We had thought that the problem of supply had been resolved during the preparation of this manuscript, but after further discussions with the company after our original submission, we were informed that stocks were not large enough to include children with CD.

### Sample size

The sample size for the proposed trial was based on a desirable increase of +0.5 HtSDS. While the desired amount of growth stimulated by medical intervention has been the subject of debate, an increase of 0.5 HtSDS is that recommended by a joint collaboration of paediatric endocrinologists across Europe and North America [17]. It was also based on efficacy of rhGH in a preliminary study of 22 children randomly allocated to receive rhGH [14]. The power calculation was based on this prior data [14] that reports a range of HtSDS of [−0.9; 2.0]. A simple, approximate, way to estimate the standard deviation from the range is by dividing the range by four which results in an estimated standard deviation of 0.725. This informs a power calculation as described by Jaki and Magirr [18] with a structure that allows for one final analysis. To detect a difference between any one dose (rhGH 0.067 or 0.035 mg/kg/day) and optimal anti-inflammatory therapy and optimal anti-inflammatory therapy alone of 0.5 HtSDS (approx. 3.5 cm) with alpha = 5% (one-sided family-wise error rate) and 80% power (1-beta = 0.8) requires recruitment of 99 participants with complete primary outcome, with 33 in each arm.

### Numbers of potential participants from a population of children with Crohn’s disease

**Fig. 2:**
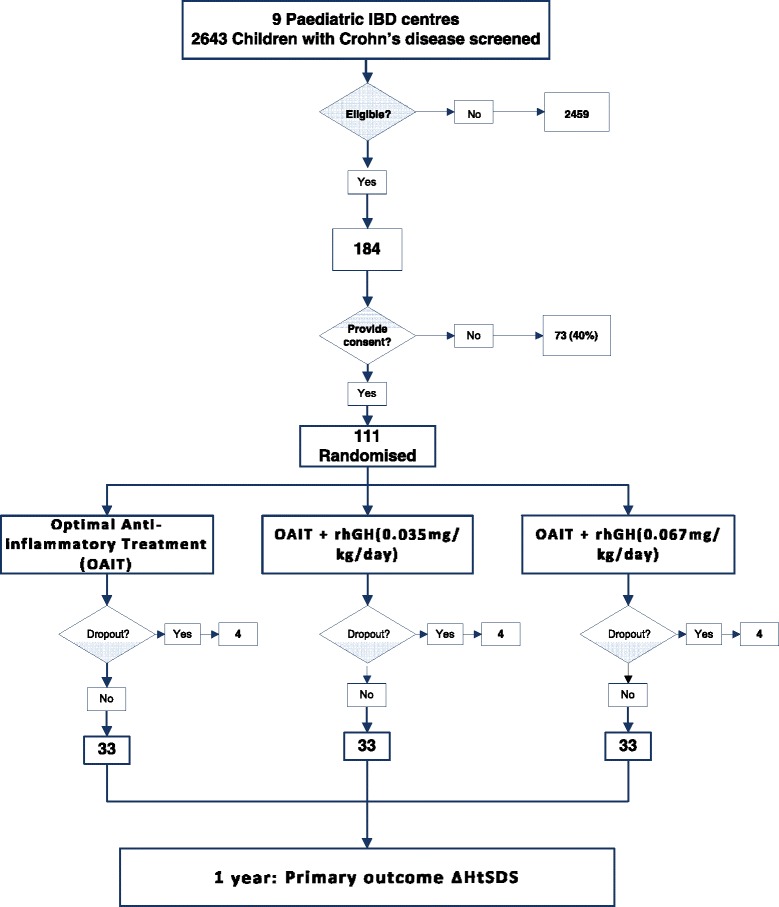
Flow diagram of the proposed future multi-centre study on the efficacy and mechanism of recombinant human growth hormone (rhGH) in children with Crohn’s disease-induced growth failure

To estimate what proportion of children with CD would be eligible for the proposed trial, we used estimates from the eligible patients at two sites (Glasgow and London) and also the results from the survey of parents and children. The databases at the two centres indicated that for every 100 children with CD, seven would fit the inclusion criteria of the study. Based on the survey data of children who were concerned about their height, 60% of these would then agree to consent and take part in the trial; thus, 4.2% of patients diagnosed with CD will be eligible. There were no dropouts in the preliminary study [14] undertaken in two of our centres. However, the MC CTU advised that it would be prudent to assume that 10% will not provide primary outcome data. We, therefore, would expect approximately 3–4 of every 100 patients with CD to be eligible, consent and provide complete primary outcome data (3.78%) (Fig. 2).

We then also estimated the numbers of patients who would present with growth failure over the first 2 years of a 3-year study. Based on Barts Health NHS Trust electronic data, for every 100 children currently attending the Inflammatory Bowel Disease clinic with Crohn’s disease, 25 new cases of CD are diagnosed each year. Assuming this ratio of new cases to prevalent cases is uniform, then for every 100 CD cases currently with growth failure, an extra 50 cases would present in the next 2 years (because the proportion of children with growth failure does not change with time, we can calculate the numbers of children who will present with growth failure from cases diagnosed in earlier years by determining the number of new cases CD in a current year).

### Sites and collaborators

We sent a letter to each paediatric gastroenterologist leading a paediatric IBD centre and to each paediatrician with an interest in gastroenterology; 14 of 18 paediatric IBD centres responded to the letter requesting the number of children with CD under their care (response rate 78%, excluding those conducting the feasibility study whose numbers we knew). No general paediatrician with an interest in gastroenterology (and therefore not working in a paediatric IBD centre) responded. The median size of each major UK paediatric IBD centre was approximately 200 [range 85–320]. If the study were to recruit over 2 years, there would be approximately 300 potentially eligible children, to include both 200 current and 100 patients presenting with growth failure in the next 2 years. To enrol 99 participants that are eligible, consent and provide complete primary outcome data in the trial, 2643 cases need to be screened (Fig. 2). Thus, approximately nine sites would be required to take part in the study to recruit the necessary participants.

## Discussion

This is the first feasibility study of an RCT in growth-promoting therapy in children with CD. It is also the first quantitative study to survey the attitude of children with CD and their parents towards endocrine therapy for growth promotion in an RCT. The possible treatments, in addition to optimal anti-inflammatory therapy (standard treatment), included daily injections of rhGH, rhIGF-1 or rhGH and rhIGF-1 in the survey. Many children with CD and their parents would take part in an RCT of growth-promoting therapy despite only a minority being very concerned about their height. However, answers may differ when confronted with consent to an ongoing trial, rather than a hypothetical one. Concerns about height were more likely in those who were shorter, and shorter children were more likely to consider this additional therapy to promote their growth.

The results of our survey can be compared with a preliminary RCT on rhGH in children with IBD [14]. In that study, all patients approached agreed to enter the study. The reasons why this proportion is so much greater include the possibility that 6 months of injections are less daunting than a year and also that the inclusion criteria in that study included a fall in height SDS (or decreased height velocity, which is the same concept differently expressed) and this made participants more keen to engage.

Reports show that boys are more vulnerable to the psychological burden of being short than are girls [19]; however, we did not find any gender difference in their concerns over height. The majority of our participants were boys (73%), and this may have influenced our results. Major reasons for not taking part in the proposed RCT reported by children in the survey were fear of injections and not being concerned about their height. Alleviating fear of injections in eligible participants may result in higher recruitment rates.

The degree of concern about height correlated with an interest in taking part in the proposed RCT, with 100% of very concerned patients willing to participate. Similarly, in a survey examining patients’ perceptions of faecal microbiota transplantation for ulcerative colitis (UC), all patients with severe UC were willing to take part [20]; thus, the patients most affected by the condition it seems are the most likely to agree to any proposed study.

Although currently, there is a lack of conclusive data on rhGH in children with IBD, the initial results suggest that rhGH may have a positive effect on growth in the short term [12–14, 21, 22]. The available evidence has shown the growth-promoting effect of rhGH on children with mild disease activity and growth retardation. Thus, the effect rhGH on CD children with intractable inflammation and growth retardation remains unanswered. There is a need to perform larger, more conclusive studies of rhGH therapy which explore this issue.

Given that substantial cohort remains short despite the use of optimal therapy and considering that the abnormality may occur at multiple levels of the GH/IGF-1 axis, the possible use of other forms of growth-promoting agents such as rhIGF-1, either alone or in combination with rhGH for promoting growth, also warrants further investigation. The studies of effects of rhIGF-1 on growth on CD have not been described yet, partly because of the theoretical risk of colon cancer in patients with high levels of circulating IGF-1. However, by using mathematical modelling to determine the dose of rhIGF-1 that could be prescribed to maintain serum IGF-1 levels within the physiological range, this study may inform the design of future clinical trials [23]. However, during the course of the feasibility study, examination of rhIGF-1 as a possible therapeutic agent had to be discontinued due to lack of supply.

One of the limitations of the survey was that the criteria for inclusion into the study did not exactly match the inclusion criteria for the proposed trial. As described above, this was because a fall in HtSDS cannot be determined anonymously. An assumption was made that those whose HtSDS had fallen by 0.5SDS would be concerned about their height. This assumption could be criticised; nevertheless, a fall of 0.5SDS in an adult male is equivalent to 4 cm, which is a change that is unlikely to go unnoticed if it happened over a year. The fact that all children in the preliminary study [14] agreed to participate leads us to conclude that 60% is not overoptimistic. A second limitation was that exploring the reasons for participants’ concern at being short was beyond the scope of a questionnaire on willingness to consent. Mason et al. [24] published the first study which showed that short stature is associated with adverse quality of life measured by IMPACT-III in the subdomain of body image. It would, therefore, be beneficial to assess the impact on quality of life in any future trial involving the use of growth-promoting therapies.

## Conclusions

In summary, while we acknowledge that, while feasible, the low proportion of children affected by growth failure will make recruitment a challenge, this study indicated that it was feasible to consider the initiation of a randomised controlled trial of an injectable form of growth-promoting therapy in children with CD. The majority of those surveyed were interested despite only a minority being very concerned about their height. By alleviating fears about injections, it is likely that a future trial would achieve the higher recruitment rates observed in the preliminary study undertaken in two of our centres [14].

## Additional files

Additional file 1: Young person’s questionnaire. (DOC 59 kb)
Additional file 2: Parent’s questionnaire. (DOC 62 kb)

## Abbreviations

CD: Crohn’s disease
GH-IGF-1: Growth hormone insulin-like growth factor-1 axis
HtSDS: Height standard deviation score
IBD: Inflammatory bowel disease
MC CTU: Medicines for Children Clinical Trials Unit
MPH: Mid-parental height
RCTs: Randomised control trials
rhGH: Recombinant human growth hormone
rhIGF-1: Recombinant human insulin-like growth factor-1
