# Supplementary material for: Assessing the feasibility of injectable growth-promoting therapy in Crohn’s disease
Source: Pilot Feasibility Stud. 2016 Dec 5;2:71. doi: 10.1186/s40814-016-0112-9 (PMC5153677; doi:10.1186/s40814-016-0112-9)
Supplement: Additional file 1: — Young person’s questionnaire. (DOC 59 kb) [file 40814_2016_112_MOESM1_ESM.doc]

**Yong Person’s Questionnaire:**

1. **About you:**
   1. How old are you? years, months *(example: 12 years, 8 months)*
   2. Are you: Male  Female
   3. Please tell us your height as measured at today’s clinic *(the clinic staff will be happy to tell you this if they haven’t already done so)*

Height centimetres Date measured: dd / mm / yyyyy

- 1. Please can you tell us your parents heights (if they haven’t completed their own form):

Mother: feet inches ( centimetres)

Father: feet inches ( centimetres)

- 1. Have you ever been treated for a growth problem? Yes

No

Don’t know

If yes, what treatment did you have:

1. **About your views:**
   1. How concerned are you about your height? Not concerned

Slightly concerned

Very Concerned

- 1. Do you think it is worth doctors trying to find a better treatment
     for growth in Crohn’s disease? Yes

No

- 1. Do you think that the opportunity of gaining extra height
      is worth a year of daily injections? Yes

No

- 1. We have explained that in an RCT you are not able to choose which
     treatment you would receive. Would you be comfortable with
     this? Yes

No

- 1. Would you be willing to attend to have your growth and other
     things checked (e.g. quality of life) if it sometimes means an extra
     clinic visit (1 or 2 extra in a year)? Yes

No

- 1. If the RCT we had in mind was happening now, would you be willing
     to join? Yes

No

You don’t have to give us a reason, but it would help us if you could provide us with more information about your response to (f) in the space below:

- 1. Has your mum or dad also completed a survey questionnaire? Yes

No

**Please add here any information that you think would be helpful to the doctors thinking about developing this study:**

***Thank you for taking the time to complete this survey. Please place the questionnaire in the envelope provided, seal the envelope and hand it in to clinic staff.***
