# Supplementary material for: Assessing the feasibility of injectable growth-promoting therapy in Crohn’s disease
Source: Pilot Feasibility Stud. 2016 Dec 5;2:71. doi: 10.1186/s40814-016-0112-9 (PMC5153677; doi:10.1186/s40814-016-0112-9)
Supplement: Additional file 2: — Parent’s questionnaire. (DOC 62 kb) [file 40814_2016_112_MOESM2_ESM.doc]

**Parent’s Questionnaire:**

1. **About you:**
   1. Are you the parent of a child with Crohn’s disease? Yes

No

(If no, please do not complete the rest of the survey)

- 1. Do you also have inflammatory bowel disease? Yes

No

- 1. Please can you tell us your height and that of the other parent:

Mother: feet inches ( centimetres)

Father: feet inches ( centimetres)

1. **About your child:**
   1. Age: years, months *(example: 12 years, 8 months)*
   2. Is your child: Male  Female
   3. Please tell us your child’s height as measured at today’s clinic *(the clinic staff will be happy to tell you this if they haven’t already done so)*

Height centimetres Date measured: dd / mm / yyyy

- 1. Has your child ever been treated for a growth problem? Yes

No

Don’t know

If yes, what treatment was given:

1. **About your views:**
   1. How concerned are you about your child’s height? Not concerned

Slightly concerned

Very Concerned

- 1. Do you think it is worth doctors trying to find a better treatment
     for growth in Crohn’s disease? Yes

No

- 1. Do you think that the opportunity of gaining extra height
     is worth a year of daily injections? Yes

No

- 1. We have explained that in an RCT you are not able to choose which
     treatment your child would receive. Would you be comfortable with
     this? Yes

No

- 1. Would you and your child be willing to attend to have your childs
     growth and other things checked (e.g. quality of life) if it sometimes
     means an extra clinic visit (1 or 2 extra in a year)? Yes

No

- 1. If the RCT we had in mind was happening now, would you be willing
     for your child to join? Yes

No

You don’t have to give us a reason, but it would help us if you could provide us with more information about your response to (f) in the space below:

- 1. Has your child also completed a survey questionnaire? Yes

No

**Please add here any information that you think would be helpful to the doctors thinking about developing this study:**

***Thank you for taking the time to complete this survey. Please place the questionnaire in the envelope provided, seal the envelope and hand it in to clinic staff.***
